# Polychlorinated Biphenyls Disrupt Intestinal Integrity via NADPH Oxidase-Induced Alterations of Tight Junction Protein Expression

**DOI:** 10.1289/ehp.0901751

**Published:** 2010-03-18

**Authors:** Yean Jung Choi, Melissa J. Seelbach, Hong Pu, Sung Yong Eum, Lei Chen, Bei Zhang, Bernhard Hennig, Michal Toborek

**Affiliations:** 1 Molecular Neuroscience and Vascular Biology Laboratory, Department of Neurosurgery and; 2 College of Agriculture, University of Kentucky, Lexington, Kentucky, USA

**Keywords:** Caco-2 cells, intestine, oxidative stress, polychlorinated biphenyls, tight junctions

## Abstract

**Background:**

Polychlorinated biphenyls (PCBs) are widely distributed environmental toxicants that contribute to numerous disease states. The main route of exposure to PCBs is through the gastrointestinal tract; however, little is known about the effects of PCBs on intestinal epithelial barrier functions.

**Objective:**

The aim of the present study was to address the hypothesis that highly chlorinated PCBs can disrupt gut integrity at the level of tight junction (TJ) proteins.

**Methods:**

Caco-2 human colon adenocarcinoma cells were exposed to one of the following PCB congeners: PCB153, PCB118, PCB104, and PCB126. We then assessed NAD(P)H oxidase (NOX) activity and expression and the barrier function of Caco-2 cells. In addition, the integrity of intestinal barrier function and expression of TJ proteins were evaluated in C57BL/6 mice exposed to individual PCBs by oral gavage.

**Results:**

Exposure of Caco-2 cells to individual PCB congeners resulted in activation of NOX and increased permeability of fluorescein isothiocyanate (FITC)-labeled dextran (4 kDa). Treatment with PCB congeners also disrupted expression of TJ proteins zonula occludens-1 (ZO-1) and occludin in Caco-2 cells. Importantly, inhibition of NOX by apocynin significantly protected against PCB-mediated increase in epithelial permeability and alterations of ZO-1 protein expression. Exposure to PCBs also resulted in alterations of gut permeability via decreased expression of TJ proteins in an intact physiological animal model.

**Conclusions:**

These results suggest that oral exposure to highly chlorinated PCBs disrupts intestinal epithelial integrity and may directly contribute to the systemic effects of these toxicants.

Polychlorinated biphenyls (PCBs) are among the most persistent and widespread environmental pollutants today. Currently, the amount of PCBs globally distributed throughout the environment is estimated at > 1.5 million metric tons (Tanabe 1988), and their bioaccumulation and bioconcentration in the food chain are well established. Indeed, the primary route of PCB exposure for most of the population is through dietary intake, with high-fish-consuming populations being at higher risk (Norström et al. 2009). Studies performed on population cohorts in the Great Lakes region consistently report that individuals with high fish consumption have a higher PCB body burden that may be related to the alterations of prenatal and postnatal cognitive development, behavior, and childhood IQ (Li et al. 2009; Stewart et al. 2008).

Very little is known about the effects of PCBs on the gastrointestinal (GI) system. We propose that exposure to PCBs through diet may influence individual health outcome via alterations of tight junctions (TJs) that seal together the adjacent intestinal epithelial cells, leading to increased intestinal permeability. The small intestine is characterized by the presence of villi that contain epithelium, which functions as a highly dynamic and selective barrier between the outside environment and underlying tissue. TJs consist of an intricate combination of transmembrane (e.g., occludin) and cytoplasmic accessory proteins [e.g., zonula occludens-1 (ZO-1)] linked to the actin cytoskeleton. The function of TJs depends on the proper expression and localization of these proteins. For example, regional loss of occludin in the intestinal epithelium was noted in an experimental model of jaundice-induced gut barrier dysfunction (Yang et al. 2005), and a decrease in ZO-1 expression was observed in hemorrhagic shock and resuscitation (Yang et al. 2003). Loss of GI epithelial integrity has been associated with the pathogenesis of several acute and chronic diseases, including diabetes, allergies, asthma, and autoimmune diseases (Liu et al. 2005).

The mechanisms of cellular and tissue toxicity of PCBs are not fully understood. However, PCBs are potent activators of oxidative stress; therefore, induction of reactive oxygen species (ROS) may be one of the mechanisms contributing to gut dysfunction. Recent evidence implicates NAD(P)H oxidase (NOX) as an important source of ROS (Harrison and Gongora 2009). Active NOX generates superoxide via one electron transfer from NADH (reduced nicotinamide adenine dinucleotide) or NADPH (nicotinamide adenine dinucleotide phosphate). Excessive activation of NOX has been implicated in the pathogenesis of inflammatory tissue injury and disease (El-Benna et al. 2009).

The aim of the present study was to evaluate the hypothesis that PCBs can disrupt gut integrity at the level of TJs. Mechanistically, our studies focused on the role of PCBs in activation of prooxidative NOX. Using *in vitro* and *in vivo* assays, we investigated whether exposure to individual PCB congeners can disrupt integrity of the intestinal epithelium via NOX-mediated disruption of TJ proteins.

## Materials and Methods

### Cell culture and PCB treatment

Caco-2 human colon adenocarcinoma cells were cultured in GlutaMax medium (Invitrogen, Carlsbad, CA) supplemented with 20% fetal bovine serum (Hyclone Laboratories, Logan, UT), 100 U/mL penicillin G, 100 μg/mL streptomycin, and 1% nonessential amino acids (all from Invitrogen). Cells were incubated with serum-free medium for 12 hr before treatment with PCB (1–10 μM) or vehicle [dimethyl sulfoxide (DMSO); control]. For NOX inhibition experiments, Caco-2 cells were pretreated for 30 min with 0.5 mM apocynin (Invitrogen), followed by exposure to individual PCB congeners. PCB126 (3,3′,4,4′,5-pentachlorobiphenyl), PCB153 (2,2′,4,4′,5,5′-hexachlorobiphenyl), PCB104 (2,2′,4,6,6′-pentachlorobiphenyl), and PCB118 (2,3′,4,4′,5-pentachlorobiphenyl) were purchased from AccuStandard (New Haven, CT).

We used PCBs at concentrations of 1–10 μM, with 5 μM used in most experiments. Such concentrations of PCBs do not affect cell viability (Eum et al. 2009) and reflect serum PCB levels in acutely exposed human populations (3.4 μM or 1 ppm) (Jensen 1989; Wassermann et al. 1979).

### Transepithelial permeability and NOX activity assays

Cells were seeded at high density (2 × 10^5^ cells/cm^2^) on fibronectin-coated Transwell tissue culture inserts (12 mm diameter, 0.4 μm pore size; Costar, Corning, NY). We assessed the transepithelial transfer of fluorescein isothiocyanate (FITC)-dextran of 4 kDa molecular weight (FD-4; Sigma Chemical Co., St. Louis, MO) as described previously (Eum et al. 2004), after cells achieved confluence and established transepithelial electrical resistance (TEER) values of 200–250 Ω × cm^2^.

NOX activity was evaluated by a lucigenin-enhanced chemiluminescence method as described previously by Sekhar et al. (2003). Intensity of chemiluminescence was measured using MikroWin2000 software and a CenrtoXS3 LB 960 Microplate Luminometer (Berthold Technologies, Oak Ridge, TN).

### Immunoprecipitation, Western blotting, and immunofluorescence

Immunoprecipitation and Western blotting were performed as described previously (Lim 2007). Protein lysates were loaded onto 10% SDS-PAGE precast gels, electrophoresed (120 V, 1 hr), and transferred onto a polyvinyl difluoride membrane (0.2 μm) at 200 mAmp for 2 hr. Membranes were blocked in 3% bovine serum albumin and incubated in respective primary antibody (1:500 dilution) overnight at 4°C. We obtained anti-ZO-1 and anti-occludin antibodies from Zymed Laboratories (Carlsbad, CA); anti-phospho-p47phox antibody from Abcam (Cambridge, MA); and anti-gp91phox (gp91 subunit) and anti-p47phox (p47 subunit) antibodies from Santa Cruz Biotechnology (Santa Cruz, CA). The next day, membranes were washed and incubated with species-specific horseradish peroxidase–conjugated secondary antibody (1:2,000 dilution; Cell Signaling Technology, Danvers, MA). Proteins of interest were detected using ECL Plus (GE Healthcare Life Sciences, Piscataway, NJ) and semiquantitated with UN-SCAN-IT gel (Silk Scientific Corp., Orem, UT, USA).

For immunofluorescence, cultures were fixed with ice-cold 4% formaldehyde for 15 min. Nonspecific binding was blocked by 10% goat serum (Sigma). Cells were washed with Tris-buffered saline (TBS) containing 0.05% Tween 20 and incubated overnight at 4°C with primary antibody diluted (1:100) in TBS. Cells were then incubated with FITC-conjugated goat anti-mouse IgG, Texas red–conjugated goat anti-rabbit IgG, or FITC-conjugated rabbit anti-goat IgG (1:1,000 dilution) for 1 hr at 25°C. Images were viewed using a confocal microscope under identical instrument settings.

### Animals

All animal protocols in this study were approved by the Committee on Animal Care at the University of Kentucky. The animals were treated humanely and with regard for alleviation of suffering. Male C57BL/6 mice (12–14 weeks of age; Charles River Laboratories, Wilmington, MA) were housed under 12:12 hr light:dark conditions with access to food and water *ad libitum*. Animals were fasted overnight before PCB treatment (150 μmol/kg). The 150-μmol/kg dose results in plasma PCB levels of 5 μM, reflecting the concentration that was used *in vitro*. Individual PCB congeners were dissolved in tocopherol-stripped safflower oil (Dyets Inc., Bethlehem, PA) and administered in a 0.1-mL volume via oral gavage using an 18-gauge gavage needle, 3 in. long, curved, 2.25 mm ball diameter (Popper and Sons, New Hyde Park, NY). Control animals received safflower oil vehicle. Mice were either sacrificed for tissue analysis or used in intestinal permeability experiments 24 hr after PCB or vehicle treatment.

### Intestinal permeability

We assessed gut permeability as described by Wang et al. (2001). Briefly, mice were anesthetized with isoflurane and administered FITC-dextran [20 kDa (FD-20); 22 mg/kg body weight in 100 μL phosphate-buffered saline] via oral gavage. Blood samples were collected at 1, 3, and 5 hr after FD-20 administration. Plasma concentrations of FD-20 were determined at 485 nm (excitation) and 530 nm (emission).

### Immunohistochemistry

Analyses were performed on 1–3 cm gastroduodenal segments, which were fixed in 10% (vol/vol) formalin, embedded in paraffin, and sectioned (6 μm) using a Finesse microtome (Shandon Cryotome, Fisher Scientific, Waltham, MA). The sections were deparaffinized, rehydrated, and stained using an adaptation of previously described methods (Pu et al. 2003).

### Statistical analysis

We used SigmaStat 2.0 software (Jandel Corp., San Rafael, CA) for statistical analysis. Comparisons between treatments were made by one-way or two-way analysis of variance followed by Tukey’s pairwise multiple comparison procedure. Statistical probability of *p* < 0.05 was considered significant.

## Results

### Individual PCB congeners induce NOX activity via phosphorylation of the p47 subunit

**Figure 1:**
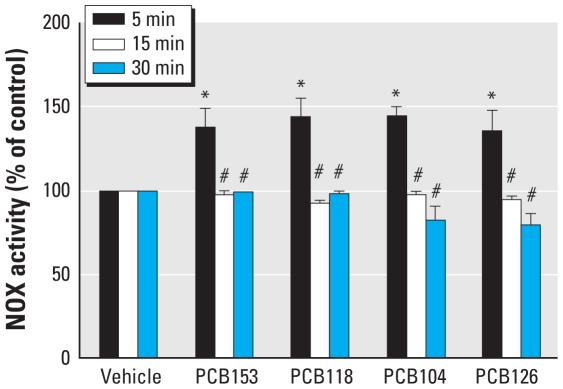
Treatment with individual PCB congeners stimulates NOX activity in Caco-2 cells. See “Materials and Methods” for details. Results are the mean ± SE (*n* = 6–7). *p < 0.05 compared with vehicle-treated control cultures. ^#^*p* < 0.05 compared with 5 min PCB treatment.

We investigated the hypothesis whether NOX-mediated reactions are involved in PCB-induced cellular toxicity. We exposed Caco-2 cells to individual PCB congeners at the concentration of 5 μM and assessed NOX activity using the lucigenin-enhanced chemiluminescence assay. All measurements were performed at the same time, immediately after the end of PCB exposure. As illustrated in Figure 1, a 5-min exposure to all studied PCBs resulted in a significant increase in NOX activity by approximately 40%. However, NOX activity was similar to control values after treatment with PCBs for 15 or 30 min.

**Figure 2:**
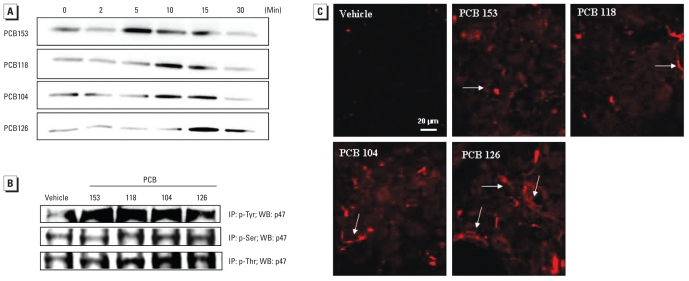
Treatment with individual PCB congeners increases the expression level and phosphorylation of the p47 NOX subunit. (*A*) p-p47 levels in Caco-2 cells exposed to DMSO (controls) or PCBs at 5 μM for up to 30 min; Western blotting (WB) was performed using whole-cell extracts immediately after the termination of PCB exposure. (*B*) p-p47 levels in Caco-2 cells exposed to PCB congeners (5 μM) for 15 min, determined by immunoprecipitation (IP) with phosphotyrosine (p-Tyr), phosphoserine (p-Ser), or phosphothreonine (p-Thr) antibody, and immunoblotting with p47 antibody. The blots in *A* and *B* are representative images from at least three experiments. (*C*) Immunofluorescence analysis of p-p47 in cells exposed to individual PCB congeners for 5 min. Arrows indicate examples of presumed membrane localization of p-p47. The images are representative data from three independent experiments.

Assembly of the active NOX complex is associated with phosphorylation of the p47 subunit and its translocation from the cytosol into the membrane fraction. Therefore, we measured p47 phosphorylation after PCB treatment of Caco-2 cells. Figure 2A indicates that exposure to all studied PCBs for 5–15 min resulted in an increase in phosphorylated p47 (p-p47) levels.

Proteins can be phosphorylated on tyrosine, serine, and/or threonine residues. Therefore, we designed additional co-immunoprecipitation experiments to determine the type of p47 phosphorylation in response to PCB exposure. Treatment with PCB congeners for 5 min increased tyrosine, serine, or threonine phosphorylation (Figure 2B), with tyrosine phosphorylation being the most prominent type of phosphorylation. Pretreatment with the NOX inhibitor apocynin (0.5 mM) appeared to inhibit all three forms of p47 phosphorylation (data not shown).

We also performed immunoreactivity experiments in Caco-2 cells treated with individual PCB congeners for 5 min (Figure 2C). Consistent with Western blotting results, we observed an increase in p-p47 immunoreactivity in all PCB-treated cultures. The staining pattern suggests localization of p-p47 in the membrane fractions of Caco-2 cells (Figure 2C).

### Treatment with individual PCB congeners stimulates p47 translocation and its association with membrane subunits of NOX

**Figure 3:**
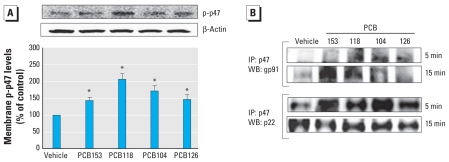
Individual PCB congeners increase the assembly of NOX complex in cellular membranes. (*A*) Expression level of p-p47 NOX determined in the membrane fraction from confluent PCB cultures treated with 5 μM of individual PCBs for 5 min, shown by immunoblotting and quantitation of the immunoblot. β-Actin was used as a control. Results are mean ± SE (*n* = 4). (*B*) Association of p47 with membrane NOX subunits gp91 and p22 in Caco-2 cells treated with individual PCB congeners (5 μM) for 5 or 15 min. Cellular extracts were immunoprecipitated using anti-p47 antibody, followed by immunoblotting with anti-gp91 or anti-p22 antibody. All blots are representative of at least four experiments. *p < 0.05 compared with vehicle-treated control cultures.

NOX activity requires not only phosphorylation of p47 but also its transfer from the cytoplasm into the membrane fraction and assembly with other NOX subunits into one complex. Therefore, we assessed these events in PCB-treated Caco-2 cells. After treatment with individual PCB congeners for 5 min, we fractionated Caco-2 cells to isolated membrane fraction. As shown in Figure 3A, exposure to individual PCBs markedly increased p-p47 levels in cellular membranes.

In addition, we evaluated the interactions between p47 and NOX subunits gp91 and p22 using immunoprecipitation approaches. Figure 3B indicates that p47 associates with gp91 within 5–15 min after treatment. We observed these effects in all PCB-exposed groups; however, the interactions between p47 and gp91 appeared to be the greatest in cells exposed to PCB118 and PCB153 for 15 min. In addition, we observed a strongly pronounced interaction between p-p47 and p22 NOX subunits at 5-min exposure for all studied PCBs (Figure 3B), further indicating the formation of the active NOX complex.

### NOX is involved in PCB-induced alterations of ZO-1 protein expression

The barrier function of gut epithelium is regulated by specialized TJ protein networks that limit passive paracellular movement molecules between adjacent epithelial cells. To determine the influence of PCBs on TJ integrity, we treated Caco-2 cells with vehicle or individual PCB congeners at concentration of 1, 5, or 10 μM for 24 hr and evaluated protein expression of ZO-1 and occludin. Occludin is a transmembrane TJ protein, whereas ZO-1 belongs to a group of so-called accessory TJ proteins that link the transmembrane proteins with the cytoskeleton.

**Figure 4:**
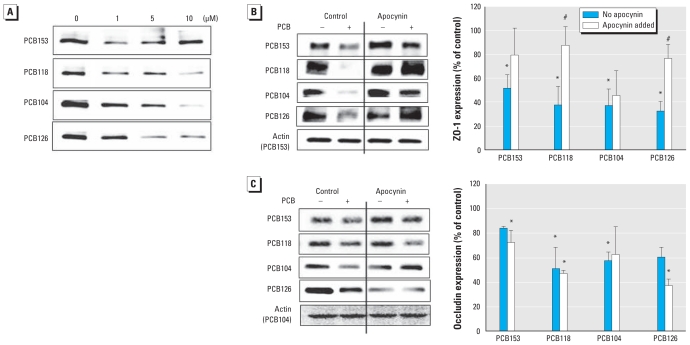
Inhibition of NOX activity attenuates the PCB-mediated decrease in ZO-1 and occludin expression in Caco-2 cells. (*A*) Expression of ZO-1 determined by Western blotting in whole-cell extracts of confluent cultures exposed to 5 μM of individual PCB congeners for 24 hr. (*B*) ZO-1 analyzed by Western blotting in confluent cells pretreated with apocynin (0.5 mM) for 30 min and then exposed to PCBs (5 μM) for 24 hr. (*C*) Western blotting analysis of occludin expression in cells treated as described for *B*. The blots in *A–C* are representative data from at least four experiments. Bar graphs in *B* and *C* represent densitometry values and statistical analysis from these experiments; in *B* and *C*, 100% represents control values. *p < 0.05 compared with vehicle-treated controls. ^#^*p* < 0.05 compared with the corresponding groups with no added apocynin.

Exposure to all PCB congeners employed in the present study resulted in a decreased expression of ZO-1 (Figure 4A). We observed these effects in Caco-2 cells exposed to individual PCBs at concentrations as low as 1 μM. To assess the role of NOX in PCB-mediated alterations of ZO-1 expression, we pretreated Caco-2 cells with apocynin, a specific NOX inhibitor, 30 min before exposure to individual PCBs. As illustrated in Figure 4B, apocynin significantly protected against a decrease in ZO-1 expression in cultures treated with PCB118 and PCB126.

In addition to alterations in ZO-1 expression, treatment with PCB congeners also decreased occludin expression in Caco-2 cells (Figure 4C). However, apocynin was not effective in protection against these effects, suggesting that NOX is not involved in PCB-induced alterations of occludin expression.

### Individual PCBs disrupt the barrier integrity of Caco-2 cells via the NOX-dependent mechanism

**Figure 5:**
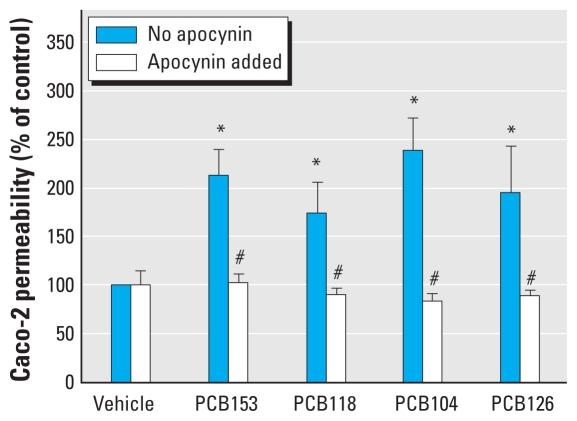
NOX inhibition protects against PCB-induced disruption of the barrier function of Caco-2 monolayers. Confluent Caco-2 cells were cultured on Transwell filters until they reached transcellular resistance of 200–250 Ω × cm^2^. They were then treated with DMSO (control) or individual PCB congeners (5 μM) for 24 hr; selected cultures were pretreated for 30 min with NOX inhibitor apocynin (0.5 mM). The barrier function of Caco-2 cells was assessed by transcellular permeability of FD-4. The results are mean ± SE (*n* = 6). *p < 0.05 compared with vehicle-treated control cultures. ^#^*p* < 0.05 compared with corresponding groups with no added apocynin.

To better understand the functional ramifications of PCB-mediated activation of NOX, the next series of our experiments focused on permeability across Caco-2 monolayers. Cultures were grown to confluence on Transwell filters until the TEER values in the range of 200–250 Ω × cm^2^ were achieved around 14–21 days after seeding. Then, cultures were exposed to individual PCB congeners at 5 μM for 24 hr. Treatment with all PCB congeners used in the present study significantly increased Caco-2 permeability to FD-4 compared with control. Importantly, the PCB-induced permeability changes were attenuated by 30-min pretreatment with the NOX inhibitor apocynin at 0.5 mM (Figure 5).

### Oral administration of PCBs alters intestinal permeability and TJ protein expression in an animal model

**Figure 6:**
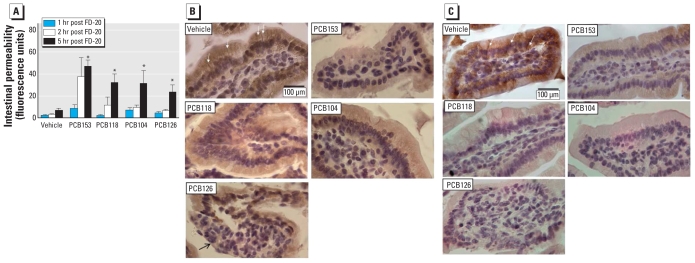
Oral administration of individual PCB congeners disrupts intestinal permeability *in vivo*. C57BL/6 mice were administered with individual PCBs (150 μmol/kg body weight) by oral gavage, and the control group received stripped safflower oil (vehicle). (*A*) Intestinal permeability assessed 24 hr after PCB administration using FD-20 that was allowed to circulate for 1, 2, or 5 hr; results are mean ± SE (*n* = 6). Immunohistochemical staining of ZO-1 (*B*) and occludin (*C*) in the small intestine of mice administered PCB congeners as described for A. Sections show individual villi; ZO-1 and occludin immunoreactivity is indicated by brown staining. In control animals, prominent ZO-1 and occludin immunoreactivity is visible at the borders of adjacent epithelial cells (*B,C*; white arrows). Administration of PCBs resulted in decreased ZO-1 and occludin expression, as indicated by the overall loss of ZO-1 and occludin staining. Administration of PCB126 markedly disrupted the morphology of villi, as indicated by loss of the villus epithelium (*B*; black arrow). *p < 0.05 compared with vehicle-treated controls.

In the last series of experiments, we employed an *in vivo* experimental model of PCB exposure in which we administered individual PCBs by oral gavage to resemble the main route of human exposure through the food chain. Mice exposed to individual PCB congeners (150 μmol/kg body weight) for 24 hr showed statistically significant increased gut permeability compared with vehicle-treated controls (Figure 6A). The assay was performed by assessing FD-20 flux from the intestinal lumen into the plasma. Elevated plasma levels of FD-20 were apparent as early as 1 hr after FD-20 administration and reached statistical significance 5 hr after FD-20 treatment.

To explain the mechanisms of PCB-induced intestinal permeability, we examined the expression of TJ proteins in villi sections of the small intestine of treated mice. The control animals were characterized by prominent immunohistochemical staining for ZO-1 (Figure 6B) and occludin (Figure 6C), indicating borders between adjacent enterocytes (arrows point to dense bars between cells in Figures 6B and C). Oral gavage of PCB153, PCB118, or PCB104 did not appear to affect the overall morphology of villi. In contrast, the morphology of villi was highly distorted in PCB126-treated animals and included areas lacking the villus epithelium. Importantly, ZO-1 and occludin staining was markedly decreased, especially at the cell–cell borders, in the small intestine of animals exposed to PCB congeners. These alterations are important because loss of TJ protein expression between adjacent enterocytes may correspond to “leaky” epithelium. Indeed, PCB-induced morphological alterations of TJs and focal loss of villous epithelium correspond to functional permeability changes observed in Figure 6A.

## Discussion

Although diet remains the primary route of PCB exposure, little is known about the influence of PCBs on gut integrity. In the present study, we demonstrate for the first time that individual PCB congeners induce NOX activity leading to TJ disruption and increased gut epithelial opening. This is an important finding, as a “leaky” intestinal mucosal barrier is associated with numerous pathologies (Liu et al. 2005) and may increase PCB entry into the body. Moreover, PCB-mediated proinflammatory and oxidative-stress–related damage to the mucosal immune system and/or intestinal epithelium may disrupt natural defenses and increase the passage of enteric pathogens across the epithelium into the circulation.

ROS and enhanced tissue oxidative stress appear to underlie the long-term toxicity of PCBs. For example, the markers of enhanced lipid peroxidation have been increased in Yusho victims 35 years after accidental poisoning with PCBs (Shimizu et al. 2007). However, the cellular sources of PCB-induced ROS are not fully understood; therefore, we explored the effects of individual PCB congeners on NOX assembly and activity. This focus was supported by the recent observation that NOX is involved in PCB-induced ROS production in neutrophils (Myhre et al. 2009). In addition, evidence from our laboratory demonstrated that PCB-induced NOX activity is required for up-regulation of cell adhesion molecules in human brain endothelial cells (Eum et al. 2009). Results of the present study show that treatment with both coplanar and non-coplanar PCBs markedly increased activity of NOX. The NOX system can be activated through several signaling pathways, including mitogen-activated protein kinases (MAPKs), protein kinase C (PKC), and Ca^2+^ (Myhre et al. 2009); thus, it is likely that different PCBs may stimulate different aspects of this pathway, resulting in final assembly of an active enzyme. Activation of NOX involves PKC-mediated phosphorylation and the recruitment of the cytosolic subunit p47 to the membrane. Functionally, p-p47 acts as an adaptor protein helping to increase the binding affinity of other NOX components, such as gp91 and p22 (Babior 2004). Consistent with these data from the literature, our results show that both coplanar PCBs (i.e., PCB126) and non-coplanar PCBs (i.e., PCB104, PCB118, and PCB153) stimulate phosphorylation of p47 and its interaction with other key NOX subunit proteins.

We next addressed the hypothesis that PCBs may act through the NOX mechanism to elicit TJ modulation within intestinal epithelium. These experiments were based on the observations that increased ROS can rapidly stimulate compartmental redistribution of occludin and ZO-1 in Caco-2 cultures (Musch et al. 2006). Studies performed in our laboratory (Zhong et al. 2008) and other (Persidsky et al. 2006) laboratories demonstrated that oxidative stress can alter the expression of TJ proteins acting through Ras and Rho redox responsive elements and activation of protein tyrosine kinase (Haorah et al. 2007). Importantly, exposure to PCBs can up- regulate activities of MAPK, phosphatidylinositol 3-kinase, or c-Src (Eum et al. 2004, 2008, 2009), that is, signaling mechanisms that may influence integrity of TJs (Rao 2009). Finally, proteolysis of TJ proteins by matrix metalloproteinases and ubiquitination- proteasome systems may also be responsible for PCB-induced changes in TJ protein expression (Eum et al. 2008).

We observed that inhibition of NOX by apocynin protected against PCB-induced alterations of ZO-1 but not occludin expression. Such a differential effect indicates finely tuned regulation of TJ expression in epithelial cells. For example, occludin phosphorylation in epithelial cells is regulated by the balance between protein kinases (e.g., c-Src, PKC zeta, PKC lambda/iota) and protein phosphatases 1 and 2A, as well as protein tyrosine phosphatase 1B (Rao 2009). Thus, this complex system of phosphorylation and dephosphorylation of occludin—rather than NOX-dependent reactions—might be the dominant mechanism regulating occludin expression.

Because *in vitro* studies cannot fully reflect the complexity of cytotoxicity of PCBs, we further investigated the role of PCBs on intestinal integrity using an intact physiological model. The experiments presented here indicate for the first time that oral exposure to PCBs can significantly increase intestinal permeability to FD-20. Moreover, this effect was associated with altered immunoreactivity for the TJ proteins ZO-1 and occludin. Disruption of gut integrity by PCBs may contribute to multiple long-term and short-term adverse effects. Initial disruption of gut integrity by PCB exposure may result in increased gut permeability to PCBs, resulting in elevated circulating levels of these toxicants. PCBs are believed to cross the gut epithelium via passive diffusion and enter into the lymphatic system (Dulfer et al. 1996). A disrupted gut barrier is likely to increase concentrations of PCBs in the systemic circulation and lead to enhanced PCB accumulation in tissues.

Another consequence of PCB-induced disruption of epithelial integrity may be an increase in gut permeability to other substances and pathogens present in the gut mucosa (Ley et al. 2006). Several gut pathogens have shown to be independently associated with alterations in TJs at the intestinal barrier (Hofman 2003). For example, an experimental model of *Escherichia coli* injection demonstrated an increase in TJ permeability associated with reorganization of the actin cytoskeleton and redistribution of TJ proteins (Qin et al. 2009). Babbin et al. (2009) recently demonstrated that compromised intestinal barrier function induced by the bacterial virulence factor lymphostatin is mediated by the Rho GTPase signaling cascade. (Additionally, high circulating levels of enteric toxins have been shown to stimulate low-level systemic inflammation. Chronic, albeit low-level, systemic inflammation has been associated with several pathologies, including vascular disorders (Pizzi et al. 2008).

## Conclusion

Results of the present study demonstrate for the first time that the intestinal epithelium is highly susceptible to oral administration of coplanar and non-coplanar PCBs. Exposure to PCBs can modulate intestinal epithelial cell functions by altering TJ protein expression and functional permeability. In addition, activation of NOX is a critical event in the signaling of PCB-induced cellular stress and cytotoxicity. Thus, our study indicates that oral exposure to coplanar or non-coplanar PCBs present a significant risk to intestinal epithelial integrity and may directly contribute to the systemic effects of these toxicants.
